# Pain Incidence, Treatment, and Associated Symptoms in Hospitalized Persons With Dementia

**DOI:** 10.1093/geroni/igaa057.2752

**Published:** 2020-12-16

**Authors:** Barbara Resnick, Marie Boltz

**Affiliations:** 1 University of Maryland School of Nursing, Baltimore, Maryland, United States; 2 Penn State University, University Park, Pennsylvania, United States

## Abstract

A better understanding of the relationships between pain and other syndromes in hospitalized persons with dementia (PWD) will help establish pain as a critical symptom to target. Secondary analyses from the Fam- FFC study describe the incidence and pharmacologic management of pain, and its association with physical function, delirium, and behavioral and psychological symptoms of dementia (BPSD). The sample (N=299) was mostly female (62%), non-Hispanic (98%), and Black (53%), with a mean age of 81.6 (SD=8.5); 166 (56%) received pain medication, whereas 40% (n=43) of 108 individuals who demonstrated pain did not receive analgesics. Regression analyses showed that, controlling for age, gender, cognition, and comorbidities, pain was associated with function (t= -.3.2, p=.001), delirium (t =5.0, p < .000), and BPSD severity (t = 2.3, p=.023). Findings suggest pain may be undertreated in hospitalized PWD but should be considered to optimize function, decrease delirium, and prevent or decrease BPSD.

